# Epidemiological Patterns and Determinants of Subclinical and Overt Hypothyroidism in a Clinical Cohort from the Hail Region, Saudi Arabia: A Retrospective Cross-Sectional and Cluster Analysis Study

**DOI:** 10.3390/clinpract16070128

**Published:** 2026-07-09

**Authors:** Tareq Nafea Alharby, Sultan Almuntashiri, Majd Habib Alshammari, Shahad Hassan Alshammari, Ahad Fhaid Alzabni, Asyah Awad Alnomassi, Hanadi Saleh Alrashidi, Saleh Alghamdi, Nafees Ahemad, Sirajudheen Anwar

**Affiliations:** 1 Department of Clinical Pharmacy, College of Pharmacy, University of Hail, Hail 81442, Saudi Arabia; tn.alharby@uoh.edu.sa (T.N.A.);; 2 Medical and Diagnostic Research Center, University of Hail, Hail 81442, Saudi Arabia; 3 Department of Pharmacology and Toxicology, College of Pharmacy, University of Hail, Hail 81442, Saudi Arabia; 4 Department of Clinical Pharmacy, Faculty of Pharmacy, Al-Baha University, Al-Baha 65779, Saudi Arabia; 5 School of Pharmacy, Monash University Malaysia, Jalan Lagoon Selatan, Petaling Jaya 46150, Selangor DE, Malaysia

**Keywords:** hypothyroidism, subclinical hypothyroidism, overt hypothyroidism, prevalence, clinical profile, thyroid-stimulating hormone, free thyroxine, Saudi Arabia

## Abstract

**Background**: Hypothyroidism is a globally prevalent disorder with variations in its occurrence. However, few studies have reported its prevalence in Saudi Arabia. This study aimed to establish the prevalence, characteristics, and predictors of subclinical and overt hypothyroidism in a clinical cohort from the Hail region of Saudi Arabia. **Methods**: A retrospective cross-sectional design was used. Electronic medical records of adults (aged ≥18 years) who underwent thyroid function testing (TSH, FT4, and FT3) at the study institution were reviewed. Participants with missing laboratory or demographic data and those with hyperthyroidism (TSH < 0.4 mIU/L) were excluded. Hypothyroidism was classified biochemically using internationally accepted TSH and FT4 thresholds. The final analysis cohort comprised 724 participants. **Results**: Of 724 participants, 471 (65.1%) were euthyroid, 234 (32.3%) had subclinical hypothyroidism, and 19 (2.6%) had overt hypothyroidism, yielding a total biochemical hypothyroidism prevalence of 34.9% (*n* = 253). Women were over-represented (89.6%), and obesity was prevalent (69%). The biochemical results confirmed that overt hypothyroidism showed significantly elevated TSH and decreased FT4 compared to subclinical hypothyroidism. There were non-significant associations between gender, BMI, and hypertension with thyroid status. Age was an independent predictor of hypothyroid status (OR = 0.983, 95% CI: 0.970–0.995, *p* = 0.008), and systolic blood pressure was also independently associated (OR = 1.011, 95% CI: 1.002–1.019, *p* = 0.016). Multiple regression showed age, hypertension, gonarthrosis, and chest pain were independently associated with TSH levels. Hierarchical clustering suggested potential regional patterns in hypothyroidism prevalence. **Conclusions**: Hypothyroidism, mostly subclinical, was prevalent in this clinical cohort. Age and systolic blood pressure were independent predictors, while BMI and gender were not. Biochemical assessment is essential for detection, and larger prospective studies are recommended.

## 1. Introduction

Hypothyroidism, a hormonal disorder, is commonly divided into two types: overt hypothyroidism, characterized by elevated thyroid-stimulating hormone (TSH) and decreased free thyroxine (FT4) levels, and subclinical hypothyroidism, characterized by elevated TSH with normal FT4 levels. This study focuses specifically on primary hypothyroidism, which is defined by elevated TSH, reflecting reduced negative feedback from the thyroid gland. Central (secondary and tertiary) hypothyroidism, caused by hypothalamic or pituitary dysfunction and characterized by low or inappropriately normal TSH with reduced FT4, is not detectable by the TSH-elevation criteria and is outside the scope of this study. The signs and symptoms range from non-specific, asymptomatic biochemical abnormalities to overt disease impacting quality of life, including fatigue, unexplained weight gain, cold intolerance, constipation, xerosis, and cognitive impairment. Hypothyroidism is also associated with cardiovascular comorbidities, including dyslipidemia, hypertension, and atherosclerosis [1,2]. The principal etiological categories of primary hypothyroidism include autoimmune thyroiditis (Hashimoto’s thyroiditis), the most common cause in iodine-sufficient populations, iatrogenic causes including thyroidectomy (*n* = 28 in this cohort) and radioiodine ablation, drug-induced hypothyroidism from agents such as amiodarone, lithium, and interferon-alpha, and congenital or genetic forms. Accurate etiological classification was not systematically available from the retrospective records which is acknowledged as a limitation. Subclinical thyroid dysfunction, including the subclinical variant, has been shown to have serious long-term consequences, such as predisposing patients to the risk of cardiovascular disease and other metabolic complications [3,4].

The global prevalence of hypothyroidism varies from 0.3% to 10%, depending on the age and sex distribution, iodine status, and methodological considerations in the studies [1]. Subclinical hypothyroidism has also been demonstrated to represent the majority of cases found in large population-based studies, showing that it is more common [2]. Female gender and age are known risk factors, with age presumably due to the autoimmune and hormonal factors intrinsic to women [3,4]. In addition to these inherent factors, lifestyle (such as obesity), iodine levels, and the presence of other conditions, such as diabetes, hypertension, and hyperlipidemia, are all known to be risk factors for the development and/or progression of thyroid disease [5,6].

Hypothyroidism is emerging as a significant public health problem in the Middle East and North Africa (MENA). This is due to urbanization, changing dietary habits, and increasing obesity, among other factors, which have all led to a shift in the epidemiology of endocrine diseases in the region [7]. In particular, Saudi Arabia has had a rapid increase in non-communicable diseases, including obesity and metabolic syndrome, which may affect thyroid function indirectly [8,9]. In the Gulf region, there have been some reports of a wide variation in the prevalence of hypothyroidism, including being greater than in Western countries [1,10]. Such variability could be the result of different populations studied, diagnostic definitions, health care, and iodine supplementation programs.

While the number of studies in this region has been growing, the prevalence of hypothyroidism in Saudi Arabia is poorly investigated. Existing studies in Saudi Arabia are limited by small samples, selected populations, and variable diagnostic criteria, creating a need for more comprehensive evaluation. These have small sample sizes, limited populations, or a lack of thorough biochemical and clinical investigations [11]. Others have limited their focus to specific populations, such as women of childbearing age, diabetics, or inpatients, thus limiting their generalizability to the population [12]. A further gap in the literature is the lack of studies that have considered the prevalence or clinical presentation of subclinical and overt hypothyroidism, as well as risk factors associated with them in an integrated framework for analysis. This is a major limitation due to absence of a holistic approach to understanding the entire spectrum of clinical and biochemical thyroid dysfunction, which is critical to ensure accurate diagnosis and clinical management of thyroid disease.

Another limitation of the literature is that different studies have identified and quantified different predictors of hypothyroidism [13]. For example, some have emphasized the importance of demographic variables (such as age and sex), but others have emphasized the importance of metabolic and cardiovascular risk factors (such as obesity and hypertension) [14]. Published studies also vary considerably in methodology, diagnostic criteria, and population selection, limiting direct comparison. These studies have found mixed results, and the strength of the association is yet to be established in the Middle East. Furthermore, few have applied advanced statistical methods (correlation and regression analyses) to determine the independent associations between a variety of co-varying factors and thyroid function variables [15]. This prevents the generation of analytical evidence to guide screening programs and disease risk models in different populations and the development of specific recommendations.

This evidence demonstrates the need for studies that offer a holistic assessment of hypothyroidism, including epidemiological, clinical, and biochemical assessment in the Saudi population. This will provide more up-to-date estimates of the prevalence but also important information about both the disease pattern and risk factor profile and potential predictors of thyroid dysfunction, which are essential in the early diagnosis, treatment, and development of strategies to address the burden of thyroid disease.

Although statistical approaches are most widely used for epidemiological studies of thyroid disease, the use of pattern recognition techniques is growing to gain insights into the variability in thyroid disease. Hierarchical clustering offers a statistical method of identifying clusters and variability in the disease (such as the proportion of subclinical and overt hypothyroidism) based on key clinical variables. This may be useful but has been rarely applied in thyroid epidemiology, particularly in comparative regional and global studies. Its application in epidemiological studies may offer additional insights into population variability and epidemiological risk factors for thyroid dysfunction [16].

The present study aimed to evaluate the prevalence, clinical characteristics, and predictors of subclinical and overt hypothyroidism in a Saudi population in the Hail region using a retrospective design. In our retrospective study, we examined the prevalence, characteristics, and correlates of subclinical and overt hypothyroidism among a group of the Saudi population. The goals of this study were to: (i) calculate the prevalence of hypothyroidism; (ii) compare the prevalence of the subclinical and overt forms of the disease; (iii) compare the clinical and laboratory characteristics of subclinical and overt hypothyroidism; and (iv) identify significant predictors and associations of thyroid dysfunction, as well as population heterogeneity, using hierarchical cluster analysis. Our global hypothesis is that subclinical hypothyroidism will be more prevalent than overt hypothyroidism, and clinical and demographic characteristics, particularly age and the presence of other diseases, will be significant correlates of thyroid dysfunction. Our study tries to address the gaps in the current literature and adds towards a more comprehensive assessment of hypothyroidism in Saudi Arabia.

## 2. Results

### 2.1. Baseline Characteristics of the Study Population

**Figure 1:**
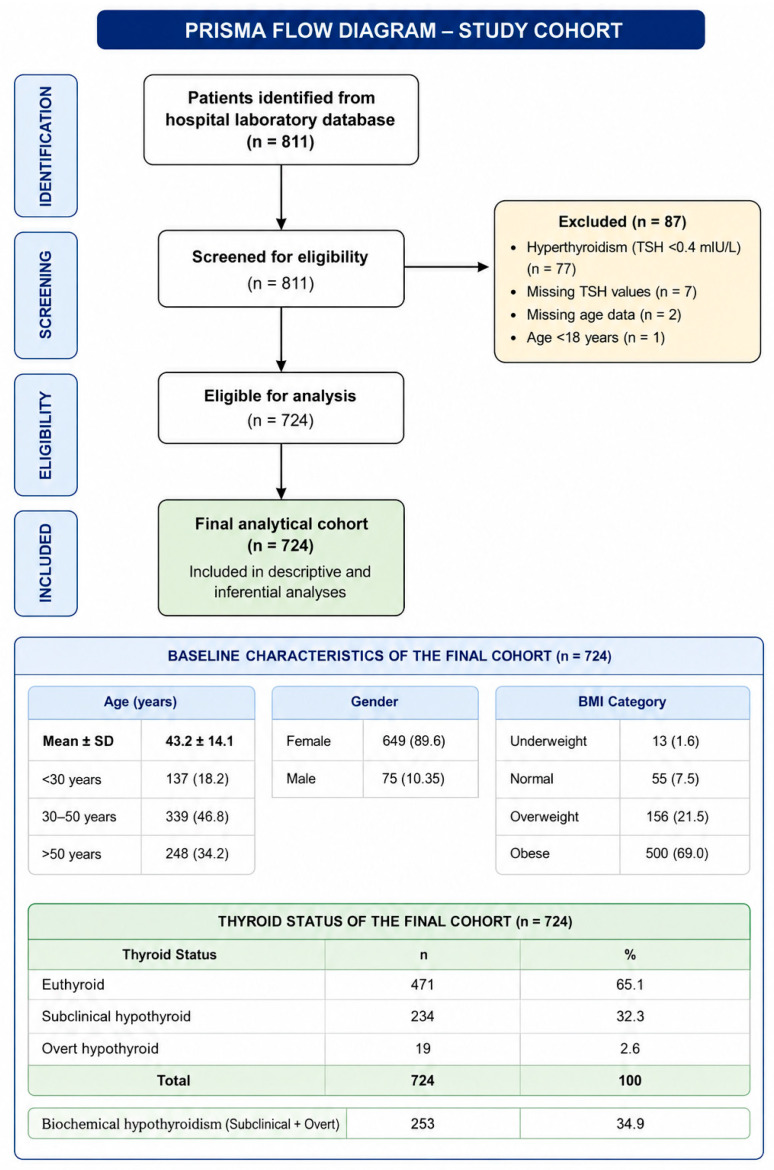
PRISMA flow diagram of participant selection and study inclusion. A total of 724 participants met the eligibility criteria and were included in the final analysis. PRISMA = Preferred Reporting Items for Systematic Reviews and Meta-Analyses.

A total of 811 patients were initially identified. After excluding 77 with hyperthyroidism (TSH < 0.4 mIU/L), 7 with missing TSH values, 2 with missing age data, and 1 aged below 18 years, the final analytical cohort comprised 724 participants. The mean age was 43.2 years (SD 14.1 years) and the majority were aged 31–50 years (46.8%). Most were women (89.6%). In terms of body mass index (BMI) the majority were obese (69%), followed by overweight (21.5%), normal weight (7.5%) and underweight (1.6%). Medians and interquartile ranges (IQR) were used to represent clinical parameters. The median heart rate was 71 bpm (IQR: 67–78), the median systolic blood pressure was 113 mmHg (IQR: 102–125) and the median diastolic blood pressure was 68 mmHg (IQR: 61–74). The most frequent comorbidities were gonarthrosis (89, 12.3%), chest pain (81, 11.2%), hypertension (58, 8.0%) and dyspepsia (9, 1.2%) (Table 1 and Figure 1).

### 2.2. Prevalence of Hypothyroidism

Thyroid status was classified biochemically using TSH and FT4 values at the time of testing. Of the 724 participants, 471 (65.1%) were euthyroid, 234 (32.3%) had subclinical hypothyroidism (TSH > 4.5 mIU/L with FT4 ≥ 11.5 pmol/L), and 19 (2.6%) had overt hypothyroidism (TSH > 4.5 mIU/L with FT4 < 11.5 pmol/L), yielding a total biochemical hypothyroidism prevalence of 34.9% (*n* = 253). Additionally, 79 participants (9.7% of the original 811) carried a prior clinical diagnosis code for hypothyroidism; of these, 44 (55.7%) were biochemically euthyroid at the time of testing, consistent with treated and stable disease on levothyroxine. A further 77 participants (9.5%) had hyperthyroidism (TSH < 0.4 mIU/L) and were excluded from the analytical cohort (Table 2).

### 2.3. Distribution of Thyroid Functional Status Among Prior-Diagnosed Participants

Among the 253 participants with biochemically confirmed hypothyroidism, subclinical disease was predominant (*n* = 234, 92.5%), with overt hypothyroidism in 19 participants (7.5%). Among the 79 participants with a prior clinical diagnosis code for hypothyroidism, 44 (55.7%) were biochemically euthyroid at the time of testing, 23 (29.1%) had subclinical hypothyroidism, and 5 (6.3%) had overt hypothyroidism, with the remainder classified as hyperthyroid or excluded (Table 3).

### 2.4. Clinical and Biochemical Characteristics by Thyroid Status

Clinical and biochemical characteristics were compared between subclinical and overt hypothyroid groups using non-parametric tests. As expected, TSH was significantly higher in overt compared to subclinical hypothyroidism (median 11.50 vs. 7.57 mIU/L; *p* = 0.003) and FT4 was significantly lower (median 9.4 vs. 13.77 pmol/L; *p* < 0.001). FT3 did not differ significantly between groups (*p* = 0.343). Cardiovascular parameters (heart rate, systolic BP, diastolic BP) did not differ significantly between thyroid status groups (Table 4).

### 2.5. Association Between Thyroid Status and Demographic Variables

A statistically significant association was observed between age group and biochemical hypothyroid status (χ^2^ = 14.974, df = 2, *p* = 0.001). The youngest age group (18–30 years) had the highest proportion of hypothyroidism (66/137, 48.2%), compared to 29.5% in the 31–50 years group (100/339) and 35.1% in the over 50 years group (87/248). This finding is consistent with the inverse association between age and hypothyroid odds identified in the multivariable logistic regression (Section 2.7). No statistically significant associations were observed between hypothyroid status and sex (χ^2^ = 0.709, *p* = 0.400), BMI category (χ^2^ = 5.072, *p* = 0.167), or hypertension (χ^2^ = 0.632, *p* = 0.427). Despite the high prevalence of obesity in this cohort (69%), obese participants did not show significantly higher odds of hypothyroidism compared to those of normal weight. Higher prevalence among female participants was similar between the hypothyroid (88.1%) and euthyroid (90.4%) groups (Table 5).

### 2.6. Correlation Analysis

**Figure 2:**
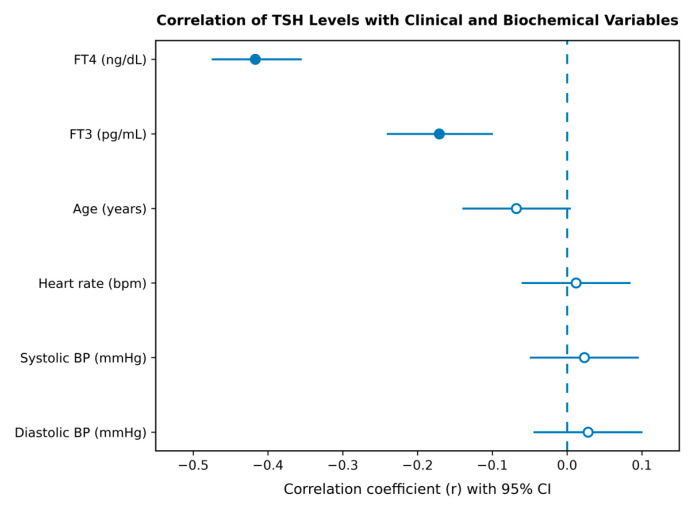
Spearman’s correlation coefficients (r) between serum TSH levels and clinical variables (*n* = 724). Points represent individual correlation coefficients and horizontal lines denote 95% confidence intervals. The vertical reference line at r = 0 indicates the null position of no correlation. Negative values reflect an inverse association with TSH. Statistical significance was set at *p* < 0.05.

Spearman’s correlation analysis revealed a moderate inverse relationship between TSH and FT4 (r = −0.417, *p* < 0.001) and a weaker inverse relationship between TSH and FT3 (r = −0.171, *p* < 0.001). Age showed no meaningful association with TSH (r = −0.067, *p* = 0.072). Cardiovascular measures were likewise unrelated to TSH levels (Table 6 and Figure 2).

### 2.7. Predictors of Hypothyroidism

**Figure 3:**
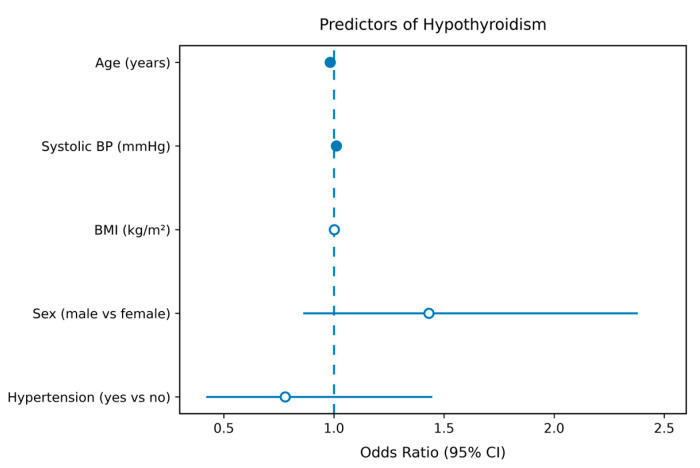
Forest plot of binary logistic regression predictors of biochemical hypothyroid status (*n* = 724). Odds ratios (OR) with 95% confidence intervals are shown. Filled circles indicate significant predictors (*p* < 0.05). Reference categories: sex = female; hypertension = absent. Model fit: χ^2^ = 12.510 (df = 5), *p* = 0.028; Nagelkerke R^2^ = 0.024; AUC = 0.568.

Binary logistic regression was applied to the full analytical cohort (*n* = 724 complete cases, 253 events, events-per-variable ratio = 50.6), with five predictors entered simultaneously: age, systolic blood pressure, BMI, sex, and hypertension status. Age emerged as an independent predictor of hypothyroid status (OR = 0.983, 95% CI: 0.970–0.995, *p* = 0.008), such that each additional year of age corresponded to a 1.7% reduction in the odds of hypothyroidism within this cohort. Systolic blood pressure also reached independent predictor status (OR = 1.011, 95% CI: 1.002–1.019, *p* = 0.016), with every 1 mmHg rise associated with a 1.1% increase in odds. The remaining three variables—BMI (OR = 1.002, *p* = 0.821), sex (OR = 1.431, *p* = 0.168), and hypertension status (OR = 0.779, *p* = 0.429)—did not reach significance in the adjusted model. The model as a whole was significant (χ^2^ = 12.510, df = 5, *p* = 0.028), yielding a Nagelkerke R^2^ of 0.024 and an AUC of 0.568. An AUC this close to 0.5 tells a clinically honest story: age and systolic blood pressure are real but weak signals, and the model carries little practical utility for stratifying individual risk (Table 7, Figure 3).

### 2.8. Predictors of TSH Levels

**Figure 4:**
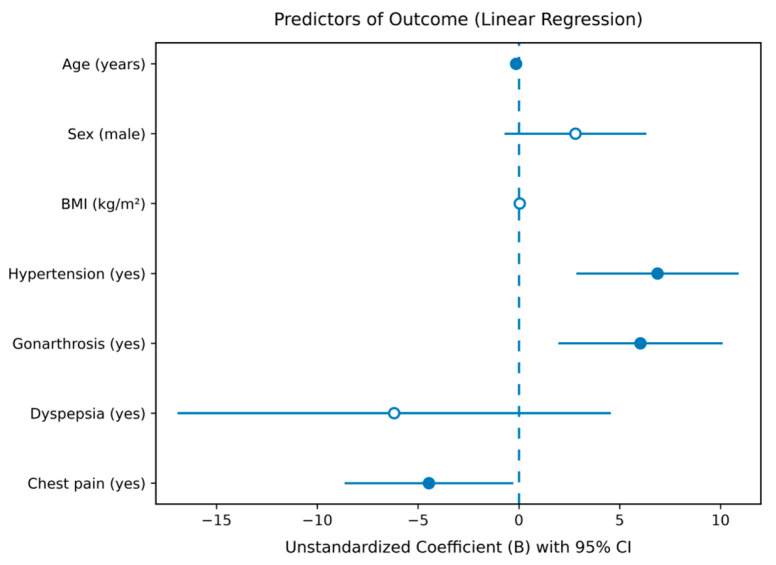
Standardized regression coefficients (β) for predictors of serum TSH levels (*n* = 724). Filled circles indicate significant predictors (*p* < 0.05). Open circles are non-significant. The vertical reference line marks β = 0. Values to the right reflect a positive association with TSH. Values to the left reflect an inverse association.

Serum TSH levels were regressed against seven clinical and demographic variables in a multiple linear regression model (*n* = 724). Four predictors reached statistical significance. Older age was associated with lower TSH (B = −0.146, β = −0.136, *p* < 0.001) a modest but consistent inverse relationship across the cohort. Participants with hypertension had markedly higher TSH on average (B = 6.874, β = 0.129, *p* < 0.001), as did those with gonarthrosis (B = 6.028, β = 0.137, *p* = 0.004). Chest pain, by contrast, was associated with lower TSH (B = −4.467, β = −0.099, *p* = 0.037). Sex, BMI, and dyspepsia contributed nothing of statistical substance to the model. Taken together, these four predictors accounted for 3.7% of the variance in serum TSH (R^2^ = 0.037, adjusted R^2^ = 0.028), which is modest but expected given that TSH is regulated principally by the hypothalamic–pituitary–thyroid axis rather than by clinical demographic factors (Table 8, Figure 4).

### 2.9. Predictors of FT4 Levels

**Figure 5:**
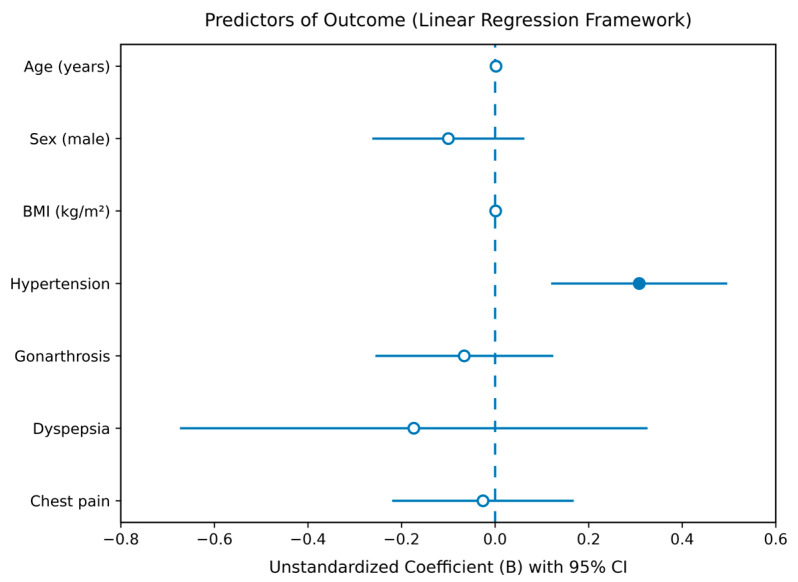
Standardized regression coefficients (β) for predictors of serum FT4 levels (*n* = 724). Filled circles indicate significant predictors (*p* < 0.05). Open circles are non-significant. The vertical reference line marks β = 0. Hypertension was the only significant independent predictor. All other variables were non-significant after adjustment.

Hypertension was the sole significant independent predictor of serum FT4 in the multivariable model (*n* = 724, R^2^ = 0.021, adjusted R^2^ = 0.012). Participants with hypertension had higher FT4 on average (B = 0.308, β = 0.125, *p* = 0.001), a finding that may reflect shared pathophysiological pathways between thyroid hormone regulation and cardiovascular function, though the cross-sectional design prevents any causal interpretation. Age showed a weak positive trend that did not reach significance (B = 0.002, *p* = 0.273). Sex, BMI, gonarthrosis, dyspepsia, and chest pain were all non-significant after adjustment (all *p* > 0.05). The low R^2^ of 0.021 confirms that the variables entered into this model account for only a small fraction of FT4 variability, which is expected given that FT4 secretion is governed primarily by pituitary TSH drive rather than by demographic or comorbid factors. The absence of a BMI effect on FT4, despite the high obesity prevalence in this cohort, is consistent with the logistic regression findings and argues against a direct adiposity-driven suppression of free thyroxine in this population (Table 9, Figure 5).

### 2.10. Hierarchical Cluster Analysis (Dendrogram)

**Figure 6:**
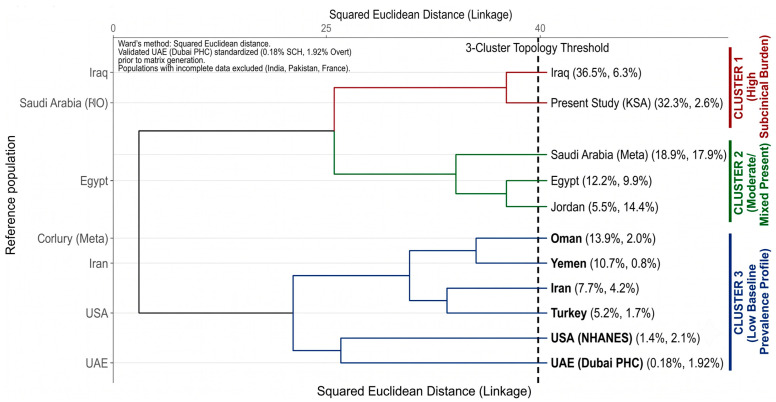
Hierarchical Clustering Dendrogram of Thyroid Profiles. SCH = Subclinical hypothyroidism; Overt = Overt hypothyroidism. Dendrogram illustrates the hierarchical clustering of 11 valid reference populations based on sample/population prevalence rates, utilizing Ward’s minimum variance linkage method and squared Euclidean distance as the dissimilarity measure. Populations with incomplete data profiles—specifically India (Amritsar), Pakistan, and France—were mathematically excluded from the cluster matrix to maintain analytical validity. To correct for reporting discrepancies, raw case proportions from the UAE (Dubai PHC) cohort were standardized to sample prevalence (0.18% subclinical, 1.92% overt) based on its 2.1% overall prevalence rate prior to matrix generation. Vertical dashed lines indicate the linkage distance thresholds establishing the optimal three-cluster topology: Cluster 1 (High Subclinical Burden), Cluster 2 (Moderate/Mixed Presentation), and Cluster 3 (Low Baseline Prevalence Profile).

Hierarchical cluster analysis was performed using Ward’s minimum variance method and squared Euclidean distance based on standardized subclinical and overt thyroid dysfunction prevalence rates. To ensure mathematical validity, the inclusion criteria required complete quantitative data for both classifications reported on a consistent population or sample prevalence basis. Out of the 14 reference populations reviewed, 11 populations met these criteria and were included in the final cluster matrix; India, Pakistan, and France were excluded due to missing values in one or both of the primary clinical metrics (Table 10). To ensure proper scaling, raw case proportions from the UAE cohort were mathematically converted to reflect sample prevalence (0.18% subclinical, 1.92% overt) using its overall reported prevalence rate (2.1%). The agglomeration schedule showed a gradual rise in fusion coefficients with a marked increase at the higher-order solutions, establishing a clean three-cluster structure that highlights regional variation (Figure 6). Cluster 1 (High Subclinical Burden): The first cluster comprised populations with a predominantly high subclinical hypothyroidism prevalence, grouping Iraq (36.5% subclinical, 6.3% overt) and the present study (Saudi Arabia) (32.3% subclinical, 2.6% overt) closely together. These cohorts share a heavily skewed distribution where early-stage or undetected subclinical disease dominates the clinical picture. Cluster 2 (Moderate/Mixed Presentation): The second cluster captured populations exhibiting intermediate or mixed epidemiological patterns where advanced progression is noted, with overt hypothyroidism rates matching or exceeding subclinical values. This branch brings together Saudi Arabia (meta-analysis) (18.9% subclinical, 17.9% overt), Egypt (12.2% subclinical, 9.9% overt), and Jordan (5.5% subclinical, 14.4% overt). Cluster 3 (Low Baseline Prevalence Profile): The third cluster grouped populations characterized by a low-to-moderate overall prevalence across both clinical presentations, with overt prevalence consistently resting under 5.0%. This expansive node brings together Oman (13.9%, 2.0%), Yemen (10.7%, 0.8%), Iran (7.7%, 4.2%), Turkey (5.2%, 1.7%), USA (NHANES) (1.4%, 2.1%), and the newly standardized UAE (Dubai PHC) (0.18%, 1.92%). Considerable variation in thyroid dysfunction prevalence was evident across the evaluated populations. Following standardization, Middle Eastern cohorts consistently separated into distinct branches based on their absolute subclinical burden rather than reporting anomalies. Our present cohort placed firmly within the high-prevalence subclinical cluster, which aligns with the total biochemical prevalence of 34.9% identified in this study. Differences in dietary iodine intake, ethnic composition, population age structure, and access to routine thyroid screening likely contribute to this regional patterning, though the analysis remains exploratory and causal inference cannot be drawn from clustering data alone (Table 10; Figure 6).

Table 10 places the findings of this study within the broader regional and global epidemiology of hypothyroidism. The diagnosis-code prevalence of 9.7% in the present cohort is comparable to estimates from the United States (11.7%) and India (9.3%), though considerably lower than the pooled prevalence of 31.3% reported in a Saudi Arabian meta-analysis. This gap most plausibly reflects differences in case definition, population selection, and TSH threshold applied across studies rather than true biological variation. The biochemical prevalence of 34.9% observed here, by contrast, aligns more closely with the meta-analytic estimate and underscores the importance of distinguishing diagnosis-code from biochemical case ascertainment when comparing prevalence figures across studies. Subclinical hypothyroidism predominated in several Middle Eastern cohorts including Iraq (36.5%) and Oman (13.9%), consistent with the pattern observed here. The UAE stands apart with overt hypothyroidism accounting for 91.4% of cases, a striking contrast that likely reflects the highly selected nature of that primary care sample rather than a genuine population-level difference. Higher prevalence among female participants was a consistent feature across cohorts, evident in Yemen (92.4%), the present study (89.5%), and India (88.0%), reinforcing the established role of sex-based hormonal and immunological factors in thyroid autoimmunity and dysfunction.

## 3. Discussion

This retrospective cross-sectional study of 724 adults in a primary care clinical cohort from the Hail region of Saudi Arabia found a biochemical hypothyroidism prevalence of 34.9%, with subclinical disease accounting for 92.5% of cases. The high prevalence reflects the clinically referred nature of the cohort: all participants had undergone thyroid function testing, enriching the sample for thyroid pathology relative to a general population screen. The diagnosis-code-based prevalence (9.7%, *n* = 79) more closely approximates figures seen in general population surveys, and the two estimates should not be conflated. Of the 79 participants carrying a prior hypothyroidism diagnosis code, 55.7% were biochemically euthyroid at the time of testing, consistent with adequately treated disease on levothyroxine [17,18]. The elevated prevalence of wo in this cohort is most plausibly attributable to the study design: participants had all undergone thyroid function testing, enriching the sample for thyroid pathology relative to an unselected population screen. The higher proportion of women and obese individuals in the cohort may have further contributed to the observed prevalence, as both female sex and obesity have been associated with thyroid dysfunction in the published literature [19,20]. Geographical variation in hypothyroidism prevalence across Middle Eastern and other populations points to the combined influence of dietary iodine intake, lifestyle factors, genetic predisposition, and differences in healthcare access and screening practices [21,22].

The high prevalence of obesity in this cohort and across the Middle East region, including the UAE, points to a potential role for metabolic factors in thyroid disease burden. Lower and less variable prevalence figures in Western populations such as the USA and France likely reflect differences in iodine sufficiency, healthcare infrastructure, and systematic screening coverage. Taken together, this comparative analysis demonstrates considerable geographical and epidemiological diversity in hypothyroidism, with subclinical disease predominating across Middle Eastern cohorts. This variation argues for population-specific screening approaches and the development of region-adapted clinical guidelines rather than uniform global thresholds [1,16,23,24,25,26,27,28,29].

The high occurrence of subclinical hypothyroidism in this cohort is consistent with published evidence identifying subclinical disease as the most common form of thyroid dysfunction [30]. The elevated prevalence likely reflects increased case detection through biochemical screening, particularly among patients presenting with non-specific symptoms or comorbid conditions. The clinical significance of subclinical hypothyroidism remains debated, though adverse cardiometabolic effects have been documented in certain populations [31]. The high prevalence observed here reinforces the case for structured monitoring and follow-up protocols for this group. Hierarchical clustering further demonstrated the considerable variability in thyroid dysfunction patterns across populations, with subclinical hypothyroidism burden emerging as the principal axis of differentiation between Middle Eastern and Western cohorts.

The use of hierarchical clustering highlighted the diversity of thyroid dysfunction across populations. Three clearly defined clusters emerged: one with high subclinical hypothyroidism prevalence, one with mixed prevalence patterns, and one with low or overt-dominant profiles. This suggests that thyroid dysfunction is not homogeneously distributed but rather reflects the specific characteristics of each population, including metabolic, iodine, genetic, and screening profiles. Middle Eastern populations consistently aligned with higher subclinical hypothyroidism prevalence, further supporting the concept of regional epidemiological clustering in thyroid disease [16]. Age was the only significant demographic predictor of hypothyroid status in the multivariable model. The inverse association (OR = 0.983 per year) indicates that younger adults in this clinical cohort had slightly higher odds of biochemically active hypothyroidism. This contrasts with population-level evidence where hypothyroidism risk increases with age [6,7], and likely reflects referral bias: younger symptomatic patients attending the thyroid clinic may be more likely to present with biochemically active disease, whereas older patients may represent a higher proportion of treated and stable cases (44 of the 79 prior-diagnosed, 55.7%, were biochemically euthyroid at testing). Systolic blood pressure showed a modest independent positive association (OR = 1.011 per mmHg), though the AUC of 0.568 confirms that the overall model has limited clinical utility for individual risk prediction. The 89.6% female composition of the cohort reflects the referral pattern of the clinic and the well-established higher prevalence and health-seeking behavior for thyroid disorders among women. These findings are most applicable to adult women in primary care settings in the Hail region and should not be generalized to the broader Saudi population without caution. Obesity was highly prevalent (69% obese by BMI ≥ 30 kg/m^2^). However, BMI was not a significant predictor in any of the three regression models (logistic: *p* = 0.821; TSH regression: *p* = 0.408; FT4 regression: *p* = 0.824). Despite the cross-sectional design precluding directional inference, this is an important negative finding. Obesity may be a metabolic consequence of hypothyroidism-related reduction in basal metabolic rate rather than a risk factor, and the absence of a significant BMI–hypothyroid association here, despite high obesity prevalence, supports the view that metabolic adiposity does not independently drive thyroid dysfunction in this cohort [8,9].

The linear regression models for TSH and FT4 identified significant but modest associations, with low variance explained (R^2^ = 0.037 and R^2^ = 0.021, respectively). Although age, hypertension, gonarthrosis, and chest pain were independently associated with TSH, and hypertension with FT4, the low R^2^ values reflect the complex physiological regulation of thyroid hormones by the hypothalamic–pituitary–thyroid axis and by factors not captured in this dataset, including iodine status, medication use, autoimmune activity, and environmental exposures [32]. he association of hypertension with both higher TSH and higher FT4 is biologically complex. Hypertension in this cohort may co-exist with metabolic syndrome affecting thyroid physiology, or may reflect a shared referral pathway rather than a direct mechanistic relationship [2]. The possibility of residual confounding from variables not included in the models cannot be excluded.

The inverse association between TSH and FT4 (r = −0.417, *p* < 0.001) confirmed the expected negative feedback relationship and supports the internal validity of the biochemical data [30]. The moderate effect size is consistent with published values in primary hypothyroid cohorts, and the scatter around this relationship reflects inherent biological variability in thyroid reserve and inter-individual assay conditions. The weaker TSH-FT3 correlation (r = −0.171) is consistent with the preferential peripheral conversion of T4 to T3, which partially maintains circulating FT3 even when TSH is mildly elevated, limiting the diagnostic sensitivity of FT3 alone in subclinical disease [8,9].

The clinical implications of these findings are that hypothyroidism, particularly subclinical disease, is prevalent in this population. The absence of strong associations with traditional risk factors such as sex, BMI, and hypertension suggests that demographic profiling alone is insufficient to guide selective screening. Broader biochemical testing in symptomatic patients, regardless of demographic profile, appears more appropriate. The inverse association between age and hypothyroid odds in this cohort, where younger patients had higher odds of active disease, argues against age-based selective screening in this particular clinical setting and instead supports systematic biochemical assessment across all adult age groups [16,29]. Broader biochemical screening across all adult age groups, rather than selective testing based on age or BMI, appears the more appropriate clinical response to the findings of this study.

The current study has several limitations. It is retrospective and cross-sectional, limiting causal inference. There is potential for selection bias since participants were drawn from a clinical thyroid service, not a random community sample; reported prevalence figures therefore reflect rates within this referred cohort and cannot be generalized to the broader Saudi population. The retrospective design did not permit systematic exclusion of conditions that may confound thyroid function, including pregnancy, active inflammatory disease, thyroid malignancy (*n* = 3), post-thyroidectomy status (*n* = 28), and use of thyroid-affecting medications such as amiodarone or lithium; these represent potential confounders that prospective studies should address. Etiological classification of hypothyroidism, such as Hashimoto’s thyroiditis or iatrogenic causes, was not systematically available from the records. The high proportions of female sex and obesity may limit generalizability to other populations. This study was adequately powered for logistic regression (EPV = 50.6), but the modest number of overt hypothyroid cases (*n* = 19) limits subgroup analyses.

Future studies should aim to include large population-based longitudinal designs incorporating a wider range of biological, environmental, and genetic variables. Longitudinal follow-up would be particularly valuable for understanding the transition from subclinical to overt hypothyroidism and for identifying early predictors of disease progression. Model performance may be improved through larger and more representative samples with external validation.

This study found a high prevalence of hypothyroidism in a clinical cohort from the Hail region of Saudi Arabia, with subclinical disease predominating. Age was a statistically significant but modest independent predictor, while traditional clinical factors including sex, BMI, and hypertension were weak or non-significant predictors of thyroid hormone levels. These findings reflect the complexity of thyroid regulation and support the case for systematic biochemical assessment rather than demographic risk-factor-based screening in clinical practice.

## 4. Materials and Methods

### 4.1. Study Design and Setting

This cross-sectional retrospective study evaluated the prevalence, clinical features, and risk factors of subclinical and overt hypothyroidism in a clinical cohort from the King Salman Specialist Hospital, Hail, Saudi Arabia. Electronic medical records of patients who underwent thyroid function testing at the study institution between February 2020 and January 2021 were reviewed. Retrospective observational designs are commonly used in epidemiology to assess disease burden and associated risk factors from routinely collected clinical data [33].

### 4.2. Study Population

A total of 811 participants with thyroid function test results were initially identified. Eligibility required age ≥ 18 years and complete results for thyroid-stimulating hormone (TSH), free thyroxine (FT4), and free triiodothyronine (FT3). The following were excluded: 77 patients with hyperthyroidism (TSH < 0.4 mIU/L), 7 with missing TSH values, 2 with missing age data, and 1 aged below 18 years. The final analytical cohort comprised 724 participants. A non-probability consecutive sampling method was adopted. Due to the retrospective design, systematic exclusion of pregnancy, active malignancy, post-thyroidectomy status, and thyroid-affecting medications were not feasible, which is acknowledged as a limitation.

### 4.3. Data Collection and Variables

Data were extracted using a standardized collection template to maintain consistency and reproducibility. Demographic variables (age, sex), anthropometric data (BMI), clinical parameters (heart rate, systolic and diastolic blood pressure), and comorbid conditions (hypertension, gonarthrosis, chest pain, dyspepsia, and all other documented diagnoses) were recorded. Biochemical measures comprised TSH, FT4, and FT3. For the purpose of analysis, age was stratified into three categories: ≤30 years, 31–50 years, and >50 years. BMI was classified according to World Health Organization criteria as underweight (<18.5 kg/m^2^), normal weight (18.5–24.9 kg/m^2^), overweight (25.0–29.9 kg/m^2^), and obese (≥30.0 kg/m^2^) [3]. Blood pressure and heart rate measurements were obtained as part of routine clinical evaluation [34].

### 4.4. Operational Definitions

Thyroid functional status was classified biochemically as follows: euthyroid (TSH 0.55–4.5 mIU/L with FT4 within the reference range (11.5–22.7 pmol/L); subclinical hypothyroidism (TSH > 4.5 mIU/L with FT4 11.5–22.7 pmol/L); overt hypothyroidism (TSH > 4.5 mIU/L with FT4 below the lower reference limit (<11.5 pmol/L); hyperthyroidism (TSH < 0.4 mIU/L, excluded from analysis). These thresholds are consistent with internationally accepted clinical practice guidelines and are commonly used in epidemiological studies [35]. Serum TSH, Free T3 (FT3), and Free T4 (FT4) were measured using the Siemens Atellica IM Analyzer(Siemens Healthcare Diagnostics Inc., Tarrytown, NY, USA). The assays were performed using the manufacturer’s original Siemens Atellica IM TSH Assay, Siemens Atellica IM FT3 Assay, and Siemens Atellica IM FT4 Assay reagent kits. These assays are based on chemiluminescent immunoassay (CLIA) technology with automated processing according to the manufacturer’s instructions [35].

### 4.5. Handling of Confounding Variables

Confounders were selected on the basis of clinical and epidemiological relevance. Correlation matrices and variance inflation factors were examined to assess multicollinearity. Variables with high intercorrelations were excluded from the models to avoid overestimation of effects.

### 4.6. Statistical Analysis

Statistical analyses were performed using SPSS version 27.0 (IBM Corp., Armonk, NY, USA) and Python 3.13 (Python Software Foundation). Normality of continuous variables was assessed using the Shapiro–Wilk test. Non-normally distributed variables were reported as median (IQR) and compared using the Mann–Whitney U test. Categorical variables were presented as *n* (%) and compared using chi-square or Fisher’s exact tests as appropriate. Spearman’s rank correlation was used to assess associations between TSH and continuous clinical variables including FT4, FT3, age, and cardiovascular parameters.

Binary logistic regression was performed on the full biochemically classified cohort (*n* = 724) to identify independent predictors of hypothyroid status, with results expressed as odds ratios (OR) with 95% confidence intervals. Predictors entered were age, systolic BP, BMI, sex, and hypertension. The events-per-variable ratio was 50.6 (253 events, 5 predictors), well above the recommended minimum of 10, confirming adequate statistical power. Model performance was assessed using Nagelkerke R^2^, model chi-square, and AUC. Multiple linear regression was performed to identify independent predictors of serum TSH and FT4 levels, entering age, sex, BMI, hypertension, gonarthrosis, dyspepsia, and chest pain as covariates. Variance inflation factors were examined to confirm absence of multicollinearity. Figures were generated using the Matplotlib (version 3.3) library in Python. A *p*-value < 0.05 was considered statistically significant throughout [36].

### 4.7. Hierarchical Cluster Analysis

Hierarchical cluster analysis was conducted to examine epidemiological patterns of thyroid dysfunction across global and regional populations. The primary input variables were the prevalence rates (%) of subclinical and overt hypothyroidism. To ensure mathematical and analytical validity, the inclusion criteria required complete quantitative data for both classifications reported on a consistent population or sample prevalence basis. Out of the 14 reference populations reviewed, 11 met these strict criteria and were included in the final cluster matrix; populations with missing entries in one or both core clinical metrics (India, Pakistan, and France) were excluded to avoid introducing imputation noise (Table 10).

Prior to analysis, metric standardization was performed on cohorts where subclinical and overt values were originally reported as a case proportion rather than sample prevalence (e.g., UAE), converting them using the cohort’s overall reported prevalence rate to guarantee a uniform metric base. Because both input variables were then natively measured on identical percentage scales, statistical data standardization (e.g., Z-score transformation) was omitted to preserve the clinical integrity of absolute prevalence differences across cohorts.

Clustering was performed using a hierarchical agglomerative algorithm with Ward’s minimum variance linkage method and squared Euclidean distance as the dissimilarity measure. Ward’s method was selected as it minimizes within-cluster variance, maximizes between-cluster distance, and is widely applied in epidemiological profiling to establish distinct, homogeneous clinical archetypes. The resulting dendrogram was examined alongside the agglomeration schedule, with substantial peaks in the fusion coefficients used to determine the optimal number of cluster partitions (Figure 6) [37].

### 4.8. Ethical Considerations

This study was conducted in accordance with the Declaration of Helsinki, and approved by the Research Ethics Committee of the University of Hail (protocol code H-2021-024) on 18 February 2021 and the Institutional Review Board, Hail Region, Saudi Arabia (protocol code H-08-L-074) on 4 March 2021. Given the retrospective nature of this study and the use of de-identified patient data, informed consent was not required. Data was kept confidential and no individual data was shared.

## 5. Conclusions

This study demonstrates a high biochemical prevalence of hypothyroidism (34.9%) in a clinical cohort from the Hail region of Saudi Arabia, with subclinical disease constituting 92.5% of cases. Age and systolic blood pressure were independent predictors of hypothyroid status, while BMI, sex, and hypertension were not. Hypertension and gonarthrosis were independently associated with higher TSH levels, and hypertension was the sole significant predictor of FT4. The absence of a significant association between obesity and hypothyroid status, despite a high obesity prevalence (69%), underscores the complexity of thyroid–metabolic interactions and the primacy of biochemical assessment over demographic profiling for case detection. Hierarchical cluster analysis placed the present Saudi Arabian cohort in a high-subclinical-hypothyroidism cluster alongside Iraq, suggesting regional epidemiological patterning that merits exploratory, prospective investigation. Larger multicenter prospective studies with standardized biochemical protocols, autoimmune markers, and longitudinal follow-up are needed.

## Figures and Tables

**Table 1:** Baseline demographic and clinical characteristics of the study population (*n* = 724).

Variable	Category	*n* (%)/Value
Age (years)	Mean ± SD	43.2 ± 14.1
	<30 years	137 (18.2)
	30–50 years	339 (46.8)
	>50 years	248 (34.2)
Gender	Female	649 (89.6)
	Male	75 (10.35)
BMI Category	Underweight	13 (1.6)
	Normal	55 (7.5)
	Overweight	156 (21.5)
	Obese	500 (69.0)
Heart Rate (bpm)	Median (IQR)	71 (67–78)
Systolic BP (mmHg)	Median (IQR)	113 (102–125)
Diastolic BP (mmHg)	Median (IQR)	68 (61–74)
Comorbidities	Gonarthrosis	89 (12.3)
	Chest pain	81 (11.2)
	Hypertension	58 (8.0)
	Dyspepsia	9 (1.2)
	Acute nasopharyngitis	5 (0.7)

BMI: body mass index, BP: blood pressure, IQR: interquartile range. All percentages use *n* = 724 as denominator.

**Table 2:** Biochemical thyroid status in the analytical cohort (*n* = 724).

Thyroid Status	*n*	%
Euthyroid	471	65.1
Subclinical hypothyroid	234	32.3
Overt hypothyroid	19	2.6
Total	724	100
Biochemical hypothyroidism	253	34.9

TSH threshold: >4.5 mIU/L. Subclinical hypothyroidism was defined as TSH > 4.5 mIU/L with FT4 within the reference range (11.5–22.7 pmol/L). Overt hypothyroidism was defined as TSH > 4.5 mIU/L with FT4 below the lower reference limit (<11.5 pmol/L).

**Table 3:** Distribution of thyroid functional status among participants with pre-diagnosed hypothyroidism (*n* = 79).

Biochemical Status at Testing	*n*	%
Biochemically Euthyroid	44	55.7
Subclinical hypothyroidism	23	29.1
Overt hypothyroidism	5	6.3
Hyperthyroid (excluded)	7	8.9
Total	79	100

Percentages reflect proportion of the 79 prior-diagnosed participants. Euthyroid status is consistent with adequately treated disease on levothyroxine.

**Table 4:** Clinical and biochemical characteristics by thyroid status among hypothyroid participants.

Variable	Subclinical Hypothyroidism Median (IQR) (*n* = 234)	Overt Hypothyroidism Median (IQR) (*n* = 19)	*p*-Value
Heart rate (bpm)	80.5 (77.0–88.0)	74 (70–92)	0.917
Systolic BP (mmHg)	128 (115–142)	119 (111–137)	0.804
Diastolic BP (mmHg)	76 (68–85)	77 (66–85)	0.945
TSH (mIU/L) *	7.57 (5.64–10.61)	11.50 (7.93–26.47)	0.003 *
FT4 (pmol/L) *	13.77 (12.23–16.47)	9.40 (8.49–9.78)	<0.001 *
FT3 (pg/mL)	2.75 (2.50–3.00)	2.83 (2.64–3.28)	0.343

* *p* < 0.05 between groups (Mann–Whitney U test). BP: blood pressure; TSH: thyroid-stimulating hormone; FT4: free thyroxine; FT3: free triiodothyronine. IQR: interquartile range.

**Table 5:** Association between biochemical thyroid status and demographic variables (*n* = 724).

Variable	Category	Hypothyroid *n* (%)	Euthyroid *n* (%)	χ^2^	*p*-Value *
Sex	Female	223 (88.1)	426 (90.4)	0.709	0.400
	Male	30 (11.9)	45 (9.6)		
Age group	18–30 years	66 (48.2)	71 (51.8)	14.974	0.001
	31–50 years	100 (29.5)	239 (70.5)		
	>50 years	87 (35.1)	161 (64.9)		
BMI category	Underweight	6 (46.2)	7 (53.8)	5.072	0.167
	Normal	26 (47.3)	29 (52.7)		
	Overweight	55 (35.3)	101 (64.7)		
	Obese	166 (33.2)	334 (66.8)		
Hypertension	Yes	17 (26.2)	41 (63.1)	0.632	0.427
	No	236 (35.4)	430 (64.6)		

* *p*-values calculated using Chi-square test. Percentages represent row proportions within each category. BMI: body mass index.

**Table 6:** Correlation of TSH levels with clinical and biochemical variables (*n* = 724).

Variable	r	*p*-Value	*n*
FT4 (pmol/L)	−0.417	<0.001	724
FT3 (pg/mL)	−0.171	<0.001	724
Age (years)	−0.068	0.066	724
Heart rate (bpm)	0.012	0.755	724
Systolic BP (mmHg)	0.023	0.538	724
Diastolic BP (mmHg)	0.028	0.446	724
BMI (kg/m^2^)	−0.035	0.353	724

Significant at *p* < 0.05. Spearman’s rank correlation used throughout due to non-normal distribution of variables. TSH: thyroid-stimulating hormone. FT4: free thyroxine. FT3: free triiodothyronine. BP: blood pressure. BMI: body mass index.

**Table 7:** Logistic regression analysis of predictors of hypothyroidism.

Variable	B	SE	*p*-Value	OR (95% CI)
Age (years)	−0.017	0.007	0.008 *	0.983 (0.970–0.995)
Systolic BP (mmHg)	0.011	0.004	0.016 *	1.011 (1.002–1.019)
BMI (kg/m^2^)	0.002	0.007	0.821	1.002 (0.988–1.016)
Sex (male)	0.358	0.260	0.168	1.431 (0.860–2.380)
Hypertension (yes)	−0.250	0.316	0.429	0.779 (0.420–1.446)

* Significant at *p* < 0.05. B: unstandardized coefficient; SE: standard error; OR: odds ratio; CI: confidence interval. Model: χ^2^ = 12.510, *p* = 0.028, Nagelkerke R^2^ = 0.024, AUC = 0.568, *n* = 724, events = 253, EPV = 50.6. Reference categories: sex = female; hypertension = absent.

**Table 8:** Multiple linear regression analysis of TSH predictors.

Predictor	B	SE	β	t	*p*-Value
Age (years)	−0.146	0.043	−0.136	−3.44	<0.001 *
Sex (male)	2.802	1.794	0.059	1.56	0.119
BMI (kg/m^2^)	0.041	0.050	0.031	0.83	0.408
Hypertension (yes)	6.874	2.055	0.129	3.34	<0.001 *
Gonarthrosis (yes)	6.028	2.079	0.137	2.90	0.004 *
Dyspepsia (yes)	−6.190	5.485	−0.042	−1.13	0.260
Chest pain (yes)	−4.467	2.135	−0.099	−2.09	0.037 *

* *p* < 0.05. B: unstandardized coefficient. β: standardized coefficient. SE: standard error. t: t-statistic. Model: R^2^ = 0.037, adjusted R^2^ = 0.028, *n* = 724. Reference categories: sex = female, hypertension = absent, gonarthrosis = absent, chest pain = absent, dyspepsia = absent.

**Table 9:** Multiple linear regression analysis of the predictors of FT4.

Predictor	B	SE	β	t	*p*
Age (years)	0.002	0.002	0.044	1.098	0.273
Sex (male)	−0.100	0.083	−0.046	−1.198	0.231
BMI (kg/m^2^)	0.001	0.002	0.008	0.223	0.824
Hypertension	0.308	0.096	0.125	3.220	0.001
Gonarthrosis	−0.066	0.097	−0.033	−0.681	0.496
Dyspepsia	−0.174	0.255	−0.026	−0.681	0.496
Chest pain	−0.026	0.099	−0.012	−0.258	0.796

Significant at *p* < 0.05. B: unstandardized coefficient. β: Standardized coefficient. SE: standard error. t: t-statistic. Model: R^2^ = 0.021, adjusted R^2^ = 0.012, *n* = 724. Reference categories: sex = female, hypertension = absent, gonarthrosis = absent, chest pain = absent, dyspepsia = absent.

**Table 10:** Epidemiological aspects of hypothyroidism in the Saudi, regional and international literature.

Country/Study	Population	Overall (%)	Subclinical (%)	Overt (%)	Female (%)	Key Notes
Present Study (Saudi Arabia)	Clinical adults *n* = 724	34.9 *	32.3 †	2.6 †	89.6	SCH predominates; obesity 69%
Saudi Arabia (meta-analysis)	General population	31.3	18.9	17.9	NR	High thyroid disorder burden
UAE (Dubai PHC)	Primary care	2.1	8.6	91.4	81.5	Overt predominance
Oman	Hospital population	NR	13.9	2.0	67.5	Moderate SCH prevalence
Yemen	Clinical cohort	NR	10.7	0.8	92.4	Significantly higher prevalence among female participants
Iran	General population	NR	7.7	4.2	NR	Balanced distribution
Iraq	General population	NR	36.5	6.3	NR	Very high SCH prevalence
Turkey	General population	NR	5.2	1.7	NR	Low prevalence
Jordan	General population	NR	5.5	14.4	NR	Higher overt proportion
Egypt	General population	NR	12.2	9.9	NR	Mixed pattern
India (Amritsar)	Urban adults	9.3	NR	NR	88	Age 31–60 predominant
Pakistan	Clinical subgroup	NR	21.5	NR	NR	Strong metabolic association
USA (NHANES)	General population	11.7	1.4–1.7	2.1	NR	Increasing prevalence trend
France	National registry	4.45	NR	NR	NR	Female: male ratio 5.6:1

* 9.7% = prior diagnosis code prevalence (*n* = 79/811). † Biochemical prevalence in analytical cohort (*n* = 724). SCH: subclinical hypothyroidism; NR: not reported; PHC: primary healthcare center. For the UAE (Dubai PHC) cohort, raw data originally reported as proportion of hypothyroid cases (8.6% subclinical, 91.4% overt) were standardized to sample prevalence (0.18% and 1.92%, respectively) based on the cohort’s overall prevalence of 2.1% to ensure comparability in cluster models.

## Data Availability

The original contributions presented in this study are included in the article. Further inquiries can be directed to the corresponding author.
